# Phenotypic and genotypic analyses to guide selection of reverse transcriptase inhibitors in second-line HIV therapy following extended virological failure in Uganda

**DOI:** 10.1093/jac/dku052

**Published:** 2014-03-14

**Authors:** R. L. Goodall, D. T. Dunn, T. Pattery, A. van Cauwenberge, P. Nkurunziza, P. Awio, N. Ndembi, P. Munderi, C. Kityo, C. F. Gilks, P. Kaleebu, D. Pillay, on behalf of the DART Virology Group and Trial Teams, P. Kaleebu, D. Pillay, P. Awio, M. Chirara, D. Dunn, D. M. Gibb, C. Gilks, R. Goodall, A. Kapaata, M. Katuramur, F. Lyagoba, R. Magala, B. Magambo, K. Mataruka, A. McCormick, L. Mugarura, T. Musunga, M. Nabankkema, J. Nkalubo, P. Nkurunziza, C. Parry, V. Robertson, M. Spyer, D. Yirrell, H. Grosskurth, P. Munderi, G. Kabuye, D. Nsibambi, R. Kasirye, E. Zalwango, M. Nakazibwe, B. Kikaire, G. Nassuna, R. Massa, K. Fadhiru, M. Namyalo, A. Zalwango, L. Generous, P. Khauka, N. Rutikarayo, W. Nakahima, A. Mugisha, J. Todd, J. Levin, S. Muyingo, A. Ruberantwari, P. Kaleebu, D. Yirrell, N. Ndembi, F. Lyagoba, P. Hughes, M. Aber, A. Medina Lara, S. Foster, J. Amurwon, B. Nyanzi Wakholi, P. Mugyenyi, C. Kityo, F. Ssali, D. Tumukunde, T. Otim, J. Kabanda, H. Musana, J. Akao, H. Kyomugisha, A. Byamukama, J. Sabiiti, J. Komugyena, P. Wavamunno, S. Mukiibi, A. Drasiku, R. Byaruhanga, O. Labeja, P. Katundu, S. Tugume, P. Awio, A. Namazzi, G. T. Bakeinyaga, H. Katabira, D. Abaine, J. Tukamushaba, W. Anywar, W. Ojiambo, E. Angweng, S. Murungi, W. Haguma, S. Atwiine, J. Kigozi, A. Latif, J. Hakim, V. Robertson, A. Reid, E. Chidziva, R. Bulaya-Tembo, G. Musoro, F. Taziwa, C. Chimbetete, L. Chakonza, A. Mawora, C. Muvirimi, G. Tinago, P. Svovanapasis, M. Simango, O. Chirema, J. Machingura, S. Mutsai, M. Phiri, T. Bafana, M. Chirara, L. Muchabaiwa, M. Muzambi, E. Katabira, A. Ronald, A. Kambungu, F. Lutwama, A. Nanfuka, J. Walusimbi, E. Nabankema, R. Nalumenya, T. Namuli, R. Kulume, I. Namata, L. Nyachwo, A. Florence, A. Kusiima, E. Lubwama, R. Nairuba, F. Oketta, E. Buluma, R. Waita, H. Ojiambo, F. Sadik, J. Wanyama, P. Nabongo

**Affiliations:** 1 MRC Clinical Trials Unit, London, UK; 2 Virco BVBA, Beerse, Belgium; 3 MRC/UVRI Uganda Research Unit on AIDS, Entebbe, Uganda; 4 Joint Clinical Research Centre, Kampala, Uganda; 5 Institute of Human Virology, Abuja, Nigeria; 6 School of Population Health, University of Queensland, Brisbane, Australia; 7 Department of Infection, University College London, London, UK; 8 Centres for Infection, Public Health England, Colindale, UK

**Keywords:** resistance, Africa, hypersusceptibility

## Abstract

**Objectives:**

We investigated phenotypic and genotypic resistance after 2 years of first-line therapy with two HIV treatment regimens in the absence of virological monitoring.

**Methods:**

NORA [Nevirapine OR Abacavir study, a sub-study of the Development of AntiRetroviral Therapy in Africa (DART) trial] randomized 600 symptomatic HIV-infected Ugandan adults (CD4 cell count <200 cells/mm^3^) to receive zidovudine/lamivudine plus abacavir (cABC arm) or nevirapine (cNVP arm). All virological tests were performed retrospectively, including resistance tests on week 96 plasma samples with HIV RNA levels ≥1000 copies/mL. Phenotypic resistance was expressed as fold-change in IC_50_ (FC) relative to wild-type virus.

**Results:**

HIV-1 RNA viral load ≥1000 copies/mL at week 96 was seen in 58/204 (28.4%) cABC participants and 21/159 (13.2%) cNVP participants. Resistance results were available in 35 cABC and 17 cNVP participants; 31 (89%) cABC and 16 (94%) cNVP isolates had a week 96 FC below the biological cut-off for tenofovir (2.2). In the cNVP arm, 16/17 participants had resistance mutations synonymous with high-level resistance to nevirapine and efavirenz; FC values for etravirine were above the biological cut-off in 9 (53%) isolates. In multivariate regression models, K65R, Y115F and the presence of thymidine analogue-associated mutations were associated with increased susceptibility to etravirine in the cABC arm.

**Conclusions:**

Our data support the use of tenofovir following failure of a first-line zidovudine-containing regimen and shed further light on non-nucleoside reverse transcriptase inhibitor hypersusceptibility.

## Introduction

Routine viral load (VL) monitoring and resistance testing to guide individual patient management is still rarely available in resource-limited settings.^1^ Switch to second-line therapy is therefore usually triggered by clinical progression, with or without the use of CD4 count measurements or a VL tiebreaker to confirm virological failure. Treatment options for first- and second-line regimens are often restricted, owing to availability and/or cost, in comparison with the individualized patient care routinely available in resource-rich settings.^2^ WHO guidelines recommend the use of standard first-line and second-line regimens, including a non-nucleoside reverse transcriptase inhibitor (NNRTI) and a boosted protease inhibitor (PI), respectively.^3^ Although tenofovir is increasingly used in first-line therapy, this remains in the minority of antiretroviral roll-out programmes to date. Triple nucleoside reverse transcriptase inhibitor (NRTI) regimens may be considered as alternative first-line treatments in special circumstances, for example in HIV-1/HIV-2 coinfection, or when specific NNRTIs may be contraindicated, not tolerated or unavailable.^3^ In the absence of individual resistance testing the selection of NRTIs to be included in the second-line regimen is problematic.^4^ The recommended drugs are based on predicted resistance patterns at failure on first-line regimens, with the rationale of minimizing potential cross-resistance.^3^ Although there are accumulating data on genotypic resistance patterns from resource-limited settings,^5,6^ phenotypic data are scarce. Given the complex resistance patterns that frequently emerge under prolonged virological failure, which is certainly more extensive than usually seen in settings with VL-determined switch to second-line therapy, phenotypic susceptibility assays are likely to provide a more accurate picture of the degree of antiviral activity provided by individual drugs.^7^

Here, we use data from NORA [Nevirapine OR Abacavir study, a sub-study of the Development of AntiRetroviral Therapy in Africa (DART) trial]^8^ to undertake a detailed drug resistance analysis of those who had virological failure after 2 years of first-line treatment with zidovudine/lamivudine plus either nevirapine or abacavir, and who did not receive virological monitoring.

## Methods

NORA^8,9^ was a randomized double-blind trial conducted in two clinical centres in Uganda as a nested sub-study within the DART trial.^10^ Six hundred previously untreated symptomatic HIV-infected adults initiating antiretroviral therapy with CD4 <200 cells/mm^3^ were randomly allocated to open-label Combivir™ (fixed-dose combination of 150 mg of lamivudine + 300 mg of zidovudine twice daily) plus blinded abacavir (300 mg twice daily; cABC arm) or nevirapine (200 mg twice daily; cNPV arm) using a double-dummy design. After 24 weeks, participants were unblinded and continued their allocated regimen with open-label drug. Although nevirapine showed short-term virological and immunological superiority over abacavir, this was not reflected in clinical outcomes.^8^ Both NORA and DART received ethics approval in Uganda [Uganda Virus Research Institute (UVRI) Science and Ethics Committee] and the UK (Imperial College). DART is registered as ISRCTN13968779. All participants provided individual written informed consent.

### Laboratory measurements

All HIV-1 RNA measurements and resistance tests were performed retrospectively. Stored plasma samples taken at baseline and 96 weeks were assayed for HIV-1 RNA using the Roche Amplicor v1.5 assay (baseline) or Roche ultrasensitive assay (week 96). Genotypic sequencing of protease and codons 1–400 of reverse transcriptase (including the connection domain) (VircoTYPE 4.3.01) and phenotypic resistance testing (Antivirogram 2.5.01, Virco BVBA)^11^ were performed on samples with HIV-1 RNA ≥1000 copies/mL at 96 weeks and on the corresponding baseline samples. Data at week 96 from participants who underwent structured treatment interruptions (from week 52 or 76)^12^ were excluded, as this intervention is likely to have had a major influence on HIV RNA levels and potentially on resistance patterns at week 96. Participants with baseline resistance or substitutions to their initial regimen (other than stavudine for zidovudine) were excluded. Phenotypic resistance was expressed as the fold-change in IC_50_ (FC) compared with wild-type (HXB2) virus for zidovudine, lamivudine, abacavir, didanosine, tenofovir disoproxil fumarate (tenofovir DF), nevirapine, efavirenz and etravirine. FC values were log_10_ transformed before analysis. Key mutations were identified by reference to the 2013 IAS–USA classification.^13^

### Statistical methods

The distribution of FC values at baseline and 96 weeks (by arm) were compared graphically. The proportions of FC values at week 96 below biological cut-offs, which represent the normal upper range in untreated patients, were calculated.^14^ The biological cut-offs used for the Antivirogram report were obtained from Virco BVBA. Changes in drug susceptibility between baseline and week 96 were examined using unpaired *t*-tests of log_10_ transformed FC values; an unpaired analysis was indicated by the weak correlation for FC values for all drugs between these two timepoints.^15^ Shifts in NNRTI FC distributions in the cABC arm (see the Results section) motivated the use of multivariate stepwise linear regression models (backwards elimination, exit probability *P* > 0.1) to identify mutations that were independently associated with 96 week FC (relative to wild-type) to nevirapine, efavirenz and etravirine. Thymidine analogue-associated mutations (TAMs) were represented as total number (0, 1–2 and ≥3) rather than as individual mutations in these models. All *P* values are two sided. All analyses were conducted in STATA version 12.1 (StataCorp LP, College Station, TX, USA).

## Results

Of the 600 participants enrolled into NORA, 300 and 300 were randomized to cABC and cNVP, respectively. Of these, 13 cABC and 19 cNVP participants died before week 96, 10 cABC and 11 cNVP participants were lost to follow-up and 37 cABC and 70 cNVP participants were randomized to a structured treatment interruption. Of the remaining 440 (240 cABC and 200 cNVP) participants, 61 (29 cABC and 32 cNVP) were no longer on their initial regimen at 96 weeks, leaving 379 (211 cABC and 168 cNVP) participants. HIV-1 RNA VL measurements were available in 363 (95.8%). A VL ≥1000 copies/mL at week 96 was seen in 58/204 (28.4%) cABC participants and 21/159 (13.2%) cNVP participants. Both a phenotypic and genotypic result was available in 38 cABC and 17 cNVP viraemic participants. Of these, 3 (all cABC) had detectable resistance mutations prior to starting therapy and were excluded, leaving 35 cABC and 17 cNVP participants available for analysis (Figure S1, available as Supplementary data at *JAC* Online). The majority of the samples were subtype A (*n* = 28; 54%), followed by subtype D (*n* = 20; 38%), A/D recombinant (*n* = 2; 4%), subtype C (*n* = 1; 2%) and C/D recombinant (*n* = 1; 2%). The distribution of subtypes was similar in the two arms (*P* = 0.4, Fisher's exact test). Stavudine was substituted for zidovudine before 96 weeks in one cABC participant. Baseline phenotypes were available in 46 cABC and 19 cNVP participants.

At week 96, the median (IQR) VL was 41 000 (8000–77 000) copies/mL in the cABC group and 33 000 (9000–98 000) copies/mL in the cNVP group. A VL ≥5000 copies/mL, the WHO virological definition of treatment failure, was seen in 26/35 (74%) cABC participants and 16/17 (94%) cNVP participants.^16^ Most participants with VL ≥1000 copies/mL at week 96 were already above this VL threshold at week 48 [18 (51%) cABC and 13 (76%) cNVP].

**Figure 1.**
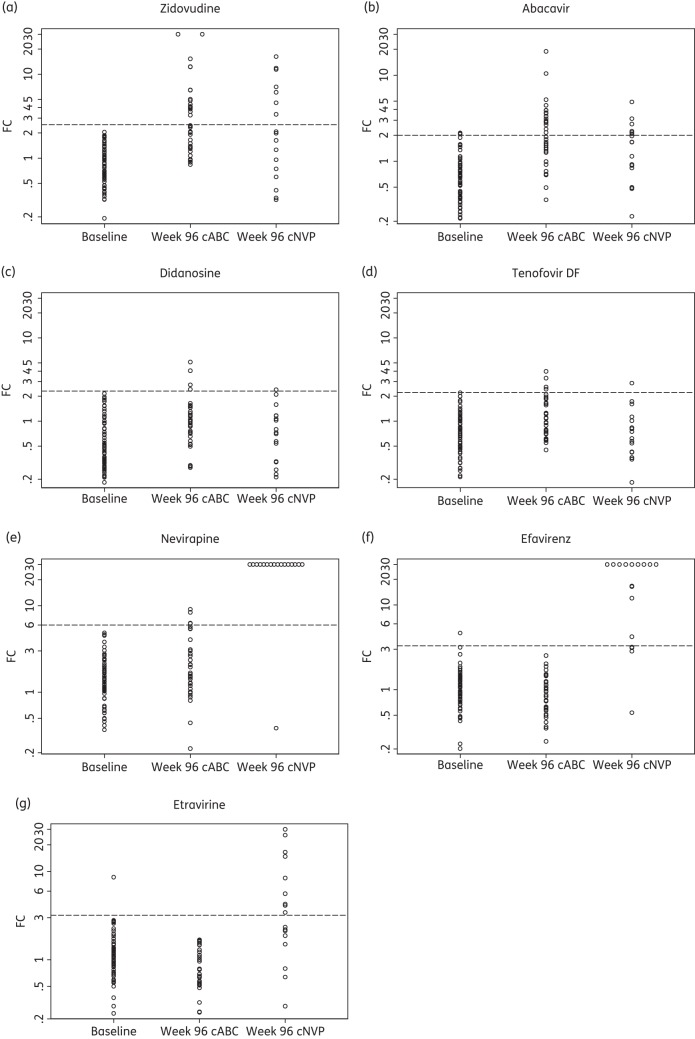
Phenotypic resistance at baseline and week 96 by antiretroviral drug. The horizontal broken line denotes the drug-specific biological cut-off. FC values >30 are displayed as 30 exactly.

The distributions of FC values for each drug are shown in Figure 1 and summarized in Table 1 in terms of the proportion of isolates with an FC below the biological cut-off and the mean proportional increase in FC compared with baseline. Lamivudine is omitted since almost all samples (30/35 cABC and 16/17 cNVP) showed very high-level resistance (median FC 62) due to the M184V mutation. FC values for zidovudine for both groups varied substantially, presumably reflecting variation in the number of TAMs in the isolates; the distribution of TAMs in each arm was as follows: 0 TAMs [8 (23%) cABC and 4 (24%) cNVP], 1–2 TAMS [9 (26%) cABC and 6 (35%) cNVP] and ≥3 TAMs [18 (51%) cABC and 7 (41%) cNVP]. Quantitatively, losses of susceptibility to didanosine and tenofovir DF were relatively small, although slightly higher for the cABC group compared with the cNVP group (*P* = 0.14 and 0.02, respectively). Most isolates had a week 96 FC value below the biological cut-off (89% for both didanosine and tenofovir DF in the cABC arm and 94% for both drugs in the cNVP arm). Many samples from participants in the cABC group had low abacavir FC values, implying continued virological residual activity from this drug.^17^ The week 96 genotypes and phenotypes for each individual are given in Table S1 (available as Supplementary data at *JAC* Online).

**Table 1.** Phenotypic resistance at week 96 by antiretroviral drug

Drug	cNVP (*n* = 17)		cABC (*n* = 35)		*P* value^b^
	*n* (%) below biological cut-off	relative change in FC^a^ (95% CI)	*n* (%) below biological cut-off	relative change in FC^a^ (95% CI)	
Zidovudine	10 (59)	2.58 (1.73, 3.86)	18 (51)	3.88 (2.86, 5.25)	0.27
Abacavir	11 (65)	2.09 (1.45, 3.00)	17 (49)	3.36 (2.51, 4.51)	0.06
Didanosine	16 (94)	1.27 (0.87, 1.83)	31 (89)	1.76 (1.33, 2.31)	0.14
Tenofovir DF	16 (94)	1.00 (0.72, 1.38)	31 (89)	1.63 (1.29, 2.06)	0.02
Nevirapine	1 (6)	29.46 (19.49, 44.54)	31 (89)	1.46 (1.10, 1.95)	<0.001
Efavirenz	4 (24)	32.47 (18.14, 58.12)	35 (100)	0.84 (0.67, 1.05)	<0.001
Etravirine	8 (47)	3.44 (2.17, 5.47)	35 (100)	0.70 (0.56, 0.89)	<0.001

^a^Ratio of geometric mean FC (relative to wild-type) at week 96 compared with geometric mean FC at week 0.

^b^*t*-test for 96 week difference in mean FC between treatment arms.

All but one participant in the cNVP arm developed a major NNRTI mutation by week 96, although most (12/16, 75%) developed a single mutation only. Individual mutations observed were G190AS (*n* = 8; 47%), Y181CV (*n* = 6; 35%), K101I (*n* = 3; 18%), A98G (*n* = 2; 12%), K103N (*n* = 2; 12%) and V108I (*n* = 1; 6%). These resulted in high-level resistance to nevirapine and efavirenz (Figure 1). FC values for etravirine were more varied than for other NNRTIs, although most (53%) of those failing on cNVP had viruses with FC to etravirine exceeding the biological cut-off of 3.2. Changes in susceptibility to all three NNRTIs were observed in the cABC arm: the average FC for nevirapine increased by 46% (95% CI: 10%–95%), but decreased for efavirenz (16%, 95% CI: −5% to 33%) and etravirine (30%, 95% CI: 11%–44%). Because no *de novo* major NNRTI mutations were observed in the cABC group (as expected) these changes are presumably due to substitutions at other positions in reverse transcriptase, including the connection domain in the C-terminal region.

To identify relevant substitutions we fitted multivariate regression models relating NNRTI FC at week 96 (relative to wild-type) to indicator variables for all mutations that were observed to develop in at least one patient in the cABC group (see the Methods section). This included three connection domain mutations, 348I, 360IV and 399D, which were present in 5 (14%) samples, 3 (9%) samples and 1 (3%) sample at week 96, respectively. No significant independent genotypic predictors were identified for nevirapine or efavirenz phenotypic resistance, although there was a trend in the expected direction for TAMs (Table 2). However, in the case of etravirine, K65R, Y115F and the presence of TAMs were associated with increased susceptibility, whilst N348I was associated with decreased susceptibility. There was no trend between the number of TAMs and etravirine FC, and the significant effect of M184V observed in the univariate analysis was lost after adjusting for the effect of the other mutations. The strength of the univariate effect of the K65R mutation was substantially reduced by the confounding effect of the presence of TAMs or the N348I mutation.

**Table 2.** Regression analysis of effect of *de novo* mutations on FC to NNRTIs (cABC arm)

Mutation	*n*^a^ (%)	Nevirapine univariate		Efavirenz univariate		Etravirine			
						univariate		multivariate^b^	
		relative change (95% CI)	*P* value	relative change (95% CI)	*P* value	relative change (95% CI)	*P* value	relative change (95% CI)	*P* value
No. of TAMs									
0	8 (23)	1.0	0.78	1.0	0.17	1.0	0.01	1.0	<0.001
1–2	9 (26)	0.87 (0.37, 2.03)		0.62 (0.37, 1.06)		0.51 (0.33, 0.80)		0.47 (0.30, 0.71)	
≥3	18 (51)	0.77 (0.37, 1.62)		0.69 (0.44, 1.10)		0.60 (0.41, 0.88)		0.49 (0.34, 0.71)	
K65R	1 (3)	1.04 (0.18, 6.03)	0.96	1.13 (0.36, 3.54)	0.83	0.99 (0.35, 2.82)	0.99	0.32 (0.12, 0.84)	0.02
69ins	1 (3)	0.53 (0.09, 3.04)	0.47	0.66 (0.21, 2.04)	0.45	0.97 (0.34, 2.76)	0.96		
L74V	1 (3)	1.31 (0.23, 7.57)	0.75	1.34 (0.43, 4.20)	0.60	1.28 (0.45, 3.61)	0.64		
Y115F	4 (11)	0.89 (0.35, 2.22)	0.79	1.10 (0.60, 2.00)	0.75	0.68 (0.40, 1.15)	0.15	0.62 (0.38, 1.00)	0.05
M184V	31 (89)	0.90 (0.36, 2.24)	0.81	0.63 (0.36, 1.13)	0.12	0.45 (0.28, 0.73)	0.002		
N348I	5 (14)	1.10 (0.48, 2.53)	0.82	1.00 (0.58, 1.73)	0.99	1.30 (0.80, 2.12)	0.28	1.77 (1.14, 2.77)	0.01
A360IV	3 (9)	2.09 (0.76, 5.75)	0.15	1.16 (0.59, 2.30)	0.65	1.01 (0.54, 1.88)	0.97		
E399D	1 (3)	0.76 (0.13, 4.38)	0.75	0.59 (0.19, 1.83)	0.35	0.65 (0.23, 1.82)	0.40		

^a^Number of patients in whom a mutation was observed at week 96 that was not observed at week 0.

^b^A backwards stepwise approach was used, starting with all variables (see the Methods section).

## Discussion

We examined phenotypic drug resistance following VL rebound after 2 years of first-line treatment, in the absence of VL monitoring, for two regimens. Zidovudine/lamivudine plus nevirapine is a commonly used first-line regimen in low- and middle-income countries,^18^ particularly with the reduction in stavudine use. Following failure with a zidovudine-containing regimen, previous WHO guidelines have stated that tenofovir and didanosine are the NRTIs most likely to have potent antiviral activity, but favour the former based on toxicity and cost considerations.^16^ However, this recommendation, as with all recommendations for second-line drugs, is based on likely viral susceptibility inferred from mutational patterns typically observed at treatment failure.^4^

Our analysis confirms that neither tenofovir nor didanosine were materially compromised by resistance that developed on this first-line regimen, with average increases in FC values of only 0% and 27%, respectively, compared with baseline. Virological response to tenofovir in treatment-experienced patients has been extensively analysed using data from two placebo-controlled intensification trials.^19^ This analysis found a weaker response with an increasing number of TAMs and decreased phenotypic susceptibility to tenofovir at baseline, although neither relationship showed a clear threshold effect. It has been argued that using tenofovir in first-line regimens and zidovudine in second-line regimens would be a more effective sequencing strategy,^20,21^ and the recently updated WHO guidelines reflect this thinking. However, even as this policy is implemented in the near future, the problem of selecting second-line drugs for patients failing on zidovudine-containing regimens will remain for many years to come. Our analysis supports the inclusion of tenofovir in second-line regimens.

Second-generation NNRTIs, including etravirine, have partially non-overlapping resistance profiles with nevirapine and efavirenz, and their utility in therapy failure in developing-world settings is of critical importance. Among participants who failed on zidovudine/lamivudine plus nevirapine, approximately one-half of the isolates had an etravirine FC of <3.2 (the biological cut-off), representing a lower bound for the proportion likely to respond successfully to the drug. However, there was substantial variability in FC values, probably reflecting the variable impact of different NNRTI mutations on etravirine resistance.^22^ Our data therefore suggest that real-time resistance testing would be necessary to establish the individualized utility of etravirine following nevirapine failure. In the absence of resistance testing, it could still play a useful role in third-line regimens in developing countries were it to become economically viable.

The other regimen used in NORA, zidovudine/lamivudine plus abacavir, is a recommended WHO first-line regimen in special circumstances, such as when the use of an NNRTI is contraindicated, not tolerated or unavailable. In practice, triple NRTI regimens are rarely used (<1% of current first-line regimens in low/middle-income countries^18^). An attractive feature of these regimens is the simplicity of constructing a potent second-line regimen including a boosted PI and an NNRTI. This leaves the quandary of the selection of NRTIs to support the PI/NNRTI combination. In this analysis, the viruses of most participants failing on zidovudine/lamivudine plus abacavir remained susceptible to didanosine and tenofovir, suggesting that either could be used. However, OHFS, a randomized DART sub-study, suggested that a two-drug, two-class, second-line regimen of lopinavir/ritonavir plus efavirenz or nevirapine was adequate for participants who failed a triple nucleoside/nucleotide first-line regimen.^23^

Although NRTIs and NNRTIs do not share any major resistance mutations, substitutions selected by NRTIs can have an impact on susceptibility to NNRTIs. Our combined genotypic and phenotypic analysis allowed exploration of this issue. Compared with baseline isolates, there was a reduction in nevirapine susceptibility, but an increased susceptibility to efavirenz and etravirine in the cABC arm; these effects were small, but statistically significant. Genotypic correlates of nevirapine and efavirenz susceptibility have been extensively studied, revealing a sensitizing effect of TAMs (both nevirapine and efavirenz)^24^ and a reduction in susceptibility (definitely nevirapine and possibly efavirenz) associated with certain connection domain mutations.^25,26^ Our regression analyses failed to identify genotypic correlates of nevirapine or efavirenz resistance, probably due to a lack of statistical power. However, we did find that the presence of TAMs, K65R and Y115F were independent predictors of increased etravirine susceptibility. Although our results are based on a small number of observations (K65R was only observed in one individual) and interpreted cautiously, the associations with TAMs and K65R have been reported previously,^27,28^ but this is, to our knowledge, the first report of an effect of Y115F. These sensitizing effects outweighed, at group level, an increase in etravirine resistance related to N348I. This association has been reported in some,^26,29^ but not all,^30^ *in vitro* studies.

A clear limitation of this study is the fact that samples were selected for VL testing at a single timepoint rather than at clinical or immunological failure. Nonetheless, our results offer an important insight into phenotypic resistance in the absence of VL monitoring, and should help inform the selection of second-line regimens in resource-limited settings.

## Funding

DART was funded by the UK Medical Research Council, the UK Department for International Development (DFID) and the Rockefeller Foundation. First-line drugs for NORA were provided by GlaxoSmithKline and Boehringer Ingelheim. Additional support for VL and resistance assays in NORA was provided by GlaxoSmithKline. This study was partly supported by the European Community's Seventh Framework Programme (FP7/2007-2013) under the project ‘Collaborative HIV and Anti-HIV Drug Resistance Network (CHAIN)’—grant agreement no. 223131.

## Transparency declarations

T. P. and A. v. C. work for Virco BVBA; they performed the genotyping and phenotying. All other authors: none to declare.

The funders had no role in study design, data collection and analysis, decision to publish or preparation of the manuscript.

### Author contributions

The NORA sub-study was conducted by P. M. and C. K., and coordinated in the UK by C. F. G. The virology sub-study was designed and coordinated by C. F. G., D. P., P. K., R. L. G. and D. T. D. The virological testing was coordinated by N. N. HIV RNA assays were carried out by P. N. and P. A. Genotyping and phenotyping were conducted by T. P. and A. v. C. Analyses were conducted by R. L. G. All authors contributed to interpretation of the data. R. L. G. wrote the first draft of the paper with D. T. D. All authors revised the manuscript critically and approved the final version. R. L. G. had full access to all the data in the study and takes responsibility for the integrity of the data, the accuracy of the data analysis and the decision to submit for publication.

## Supplementary data

Figure S1 and Table S1 are available as Supplementary data at *JAC* Online (http://jac.oxfordjournals.org/).

Supplementary Data
