# Supplementary material for: Phenotypic and genotypic analyses to guide selection of reverse transcriptase inhibitors in second-line HIV therapy following extended virological failure in Uganda
Source: J Antimicrob Chemother. 2014 Mar 14;69(7):1938–44. doi: 10.1093/jac/dku052 (PMC4054985; doi:10.1093/jac/dku052)
Supplement: Supplementary Data [file supp_69_7_1938__index.html]

Phenotypic and genotypic analyses to guide selection of reverse transcriptase inhibitors in second-line HIV therapy following extended virological failure in Uganda — Phenotypic and genotypic analyses to guide selection of reverse transcriptase inhibitors in second-line HIV therapy following extended virological failure in Uganda — Supplementary Data 

# Phenotypic and genotypic analyses to guide selection of reverse transcriptase inhibitors in second-line HIV therapy following extended virological failure in Uganda

## Supplementary Data

Supplementary Data

**Files in this Data Supplement:**

- Supplementary Data - Docx file
