# Supplementary material for: Phenotypic and genotypic analyses to guide selection of reverse transcriptase inhibitors in second-line HIV therapy following extended virological failure in Uganda
Source: J Antimicrob Chemother. 2014 Mar 14;69(7):1938–44. doi: 10.1093/jac/dku052 (PMC4054985; doi:10.1093/jac/dku052)
Supplement: Supplementary Data [file supp_dku052_dku052supp.docx]

**Supplementary data**

**Figure S1.**

**Table S1.** Individuals Week 96 Phenotype and Genotype s

| NVP |  |  |  |  |  |  |  |
| --- | --- | --- | --- | --- | --- | --- | --- |
| AZT FC | ABC FC | ddI FC | TDF FC | NVP FC | EFV FC | ETV FC | Mutations |
| 0.32 | 0.83 | 0.7 | 0.43 | 57.29 | 3.11 | 0.3 | 184V 190A 210W 215Y |
| 16.17 | 4.89 | 2.1 | 1.72 | 47.89 | 16.73 | 16.51 | 181C 184V 210W 215Y 41L |
| 7.06 | 2.16 | 2.38 | 1.61 | 66.91 | 4.22 | 4.28 | 181C 184V 210W 215F 41L 67N |
| 0.59 | 0.49 | 1.16 | 0.35 | 52.87 | 268.37 | 1.88 | 101E 184V 190A 215Y 41L |
| 1.62 | 2.23 | 0.72 | 0.42 | 50.9 | 51 | 1.5 | 103N 184V 219Q 67N 70R |
| 0.74 | 0.23 | 0.26 | 0.74 | 0.38 | 0.53 | 0.79 |  |
| 11.4 | 2.7 | 1.02 | 1.01 | 57.29 | 126.51 | 0.64 | 184V 190S 210W 215Y 41L 67N |
| 1.26 | 1.14 | 0.33 | 0.36 | 57.29 | 143.28 | 2.34 | 101E 184V 190A |
| 6.1 | 3.12 | 0.8 | 0.81 | 50.9 | 178.03 | 2.12 | 184V 190A 215F 219E 67N 70R |
| 4.57 | 1.95 | 0.54 | 0.62 | 57.29 | 107.8 | 3.44 | 184V 190A 210W 215Y |
| 0.33 | 0.48 | 1.07 | 0.36 | 52.87 | 2.83 | 14.82 | 181C 184V 70R |
| 1.98 | 0.92 | 0.21 | 0.54 | 50.9 | 3.18 | 25.72 | 181V 184V 215F |
| 0.95 | 0.89 | 0.57 | 0.57 | 57.29 | 356.77 | 4.12 | 101E 184V 190A |
| 2.07 | 1.67 | 1.16 | 1.13 | 47.89 | 557.29 | 2.19 | 103N 184V 215F 219E 67N 70R |
| 0.41 | 0.5 | 0.23 | 0.18 | 47.89 | 372.11 | 5.59 | 184V 190A |
| 3.33 | 2.08 | 1.58 | 2.86 | 66.91 | 16.4 | 133.63 | 181C 184V 215F 41L |
| 11.83 | 1.66 | 0.32 | 0.83 | 50.9 | 12.04 | 8.42 | 108I 181C 184V 210W 215Y 41L 67N |
| ABC |  |  |  |  |  |  |  |
| AZT FC | ABC FC | ddI FC | TDF FC | NVP FC | EFV FC | ETV FC | Mutations |
| 1.9 | 2.65 | 0.49 | 0.7 | 5.73 | 1.13 | 0.96 | 184V 67N 70R |
| 0.94 | 3.1 | 2.41 | 1.09 | 2.1 | 0.96 | 0.79 | 184V 65R |
| 2.07 | 5.19 | 0.71 | 0.71 | 0.92 | 0.96 | 0.51 | 115F 184V 219Q 70R |
| 0.84 | 0.69 | 0.52 | 0.89 | 5.45 | 1.87 | 1.55 | 184V |
| 1.29 | 10.46 | 4.04 | 2.08 | 2.63 | 1.14 | 1.01 | 115F 184V 74V |
| 1.49 | 0.76 | 0.52 | 0.58 | 0.98 | 1.06 | 1.68 |  |
| 4.13 | 1.44 | 0.58 | 1.23 | 5.41 | 1.53 | 1.23 | 184V 219E 67N 70R |
| 6.52 | 3.92 | 1.13 | 1.08 | 1.53 | 0.75 | 0.63 | 184V 210W 215Y 41L 67N |
| 15.26 | 2.36 | 0.99 | 1.21 | 1.26 | 0.6 | 0.78 | 184V 215F 219E 41L 67N 70R |
| 36.96 | 18.94 | 5.14 | 3.95 | 1.09 | 0.57 | 0.77 | 115F 184V 210W 215F 215Y 69SIns |
| 0.9 | 0.49 | 0.29 | 0.78 | 5.48 | 1.71 | 1.65 |  |
| 3.99 | 3.59 | 1.63 | 0.79 | 0.8 | 0.25 | 0.26 | 184V 215F 67N 70R |
| 5.03 | 3.35 | 1.45 | 2.43 | 1.01 | 0.41 | 0.69 | 184V 215F 219E 41L 70R |
| 1.34 | 0.99 | 1.51 | 0.95 | 1.49 | 0.83 | 1.08 | 184V 67N 70R |
| 1.07 | 1.59 | 0.53 | 0.76 | 1.42 | 0.63 | 0.66 | 184V |
| 12.23 | 2.36 | 1.05 | 1.66 | 3 | 1.46 | 1.13 | 184V 215F 219E 67N 70R |
| 2.02 | 1.31 | 0.73 | 0.73 | 0.88 | 0.35 | 0.48 | 184V 215Y |
| 2.33 | 1.26 | 1.54 | 1.22 | 6.3 | 0.87 | 0.57 | 184V 215F 41L |
| 2.45 | 1.38 | 0.94 | 1.62 | 1.67 | 1.13 | 1.3 | 184V 215Y 219E 67N 70R |
| 3.26 | 2.94 | 0.86 | 1.56 | 1.19 | 0.37 | 0.66 | 184V 215Y 67N 70R |
| 0.89 | 1.66 | 1.18 | 0.55 | 4.06 | 1.22 | 0.25 | 115F 184V 215F |
| 39.35 | 3.89 | 1.13 | 3.28 | 3.12 | 0.73 | 0.33 | 184V 210W 215Y 41L 67N |
| 4.09 | 3.65 | 1.13 | 0.62 | 2.36 | 0.5 | 1.01 | 184V 215Y |
| 6.47 | 1.78 | 2.73 | 1.87 | 9.06 | 2.53 | 1.01 | 184V 215F 219E 67N 70R |
| 0.97 | 0.7 | 0.65 | 0.59 | 1.61 | 1.48 | 1.47 |  |
| 3.57 | 2.82 | 0.89 | 1.1 | 8.39 | 1.47 | 0.54 | 184V 215F 219E 67N 70R |
| 2.41 | 1.53 | 1 | 0.6 | 6.21 | 2.02 | 1.47 | 184V 219E 67N 70R |
| 1.64 | 0.92 | 0.58 | 0.77 | 1.53 | 0.62 | 0.57 | 184V 67N 70R |
| 4.85 | 3.32 | 1.23 | 1.84 | 0.44 | 0.47 | 0.64 | 184V 210W 215Y 41L 67N |
| 6.51 | 2.9 | 0.87 | 2.57 | 1.54 | 0.52 | 0.52 | 184V 210W 215Y 225H 41L |
| 4.38 | 2.16 | 0.76 | 0.7 | 2.08 | 1.25 | 0.55 | 184V 210W 215F 41L 67N |
| 3.85 | 1.92 | 1.17 | 1.25 | 0.22 | 0.61 | 0.78 | 184V 219E 67N 70R |
| 1.2 | 0.71 | 0.29 | 0.45 | 1.28 | 0.58 | 1.01 | 184V 219Q 67N 70R |
| 1.39 | 0.36 | 0.28 | 0.97 | 2.78 | 1.01 | 1.61 |  |
| 12.31 | 4.46 | 1.3 | 1.95 | 1.95 | 0.95 | 1.01 | 184V 210W 215Y 41L 67N 70R |

AZT = zidovudine, ABC = abacavir, ddI = didanosine, TDF = tenofovir DF, NVP = nevirapine, EFV = efavirenz, ETV = etravirine, FC = fold-change in IC_50_.
